# *In Vitro* Use of Cellular Synthetic Machinery for Biosensing Applications

**DOI:** 10.3389/fphar.2019.01166

**Published:** 2019-10-15

**Authors:** Kyung-Ho Lee, Dong-Myung Kim

**Affiliations:** Department of Chemical Engineering and Applied Chemistry, Chungnam National University, Daejeon, South Korea

**Keywords:** cell-free synthesis, bioanalysis, *in vitro* transcription, *in vitro* translation, biosensor, microbial biosensor

## Abstract

The application of biosensors is expanding in diverse fields due to their high selectivity and sensitivity. Biosensors employ biological components for the recognition of target analytes. In addition, the amplifying nature of biosynthetic processes can potentially be harnessed to for biological transduction of detection signals. Recent advances in the development of highly productive and cost-effective cell-free synthesis systems make it possible to use these systems as the biological transducers to generate biosensing signals. This review surveys recent developments in cell-free biosensors, focusing on the newly devised mechanisms for the biological recognition of analytes to initiate the amplification processes of transcription and translation.

## Introduction

A biosensor is an analytical device that uses a biomolecule to recognize target analytes. The high specificity of biomolecules such as enzymes, antibodies, and nucleic acids comprises the unique advantage of biosensors over other assay methods. A biosensor assay readout is generated when a bio-recognition event is converted by a transducer into a readable signal. In nature, a number of fluorogenic and luminogenic proteins have suitable properties for optical measurement, including green fluorescent protein (GFP) and firefly luciferase (Bolbat and Schultz, 2017). Because measurement of luminescence and fluorescence provides instrumental compactness, selectivity, sensitivity, and assay flexibility, it is advantageous to use the exogenous expression of these proteins as a transducer of the bio-recognition event of a biosensor in order to complete the biosensing mechanism. Due to the complexity of gene expression, biosensors based on synthesis of the proteins have generally been built by introducing the gene constructs into microbial cells, comprising microbial biosensors (Raut et al., 2012; Liu et al., 2014; Gui et al., 2017). Compared with biosensors based on purified biomolecules, the co-existence of translational machinery that serves as a transducer enables engineered microbial sensors to work as self-standing devices capable of recognizing target analytes and producing signals. In particular, the modularity of gene expression allows a significant opportunity in facile design of microbial sensors that can be used for detection of a wide variety of analytes. In these systems, detection signals are generated by fusing signal-generating genes with separate sequence elements that can recognize and trigger the events of gene expression. In this approach of microbial sensors, the reporter gene and translational machinery together serve as a universal transducer to generate readable signals under the control of the target-recognizing upstream sequence of DNA, which is readily switchable for different target analytes. Recent advances in genetic engineering and synthetic biology have increased the availability of sequence elements that regulate gene expression in response to specific target molecules, and diverse recognition elements can be fused to the reporter genes systematically, accelerating the development of novel microbial sensors.

However, the widespread application of microbial sensors has been restricted by the intrinsic limitations of microbial sensors that use live cells. For example, whole cell-based microbial sensors may have a limited detection range due to analyte toxicity or membrane impermeability (Pellinen et al., 2004). More importantly, the requirement for time-consuming cell culture and conditioning steps substantially restrains the practical application of microbial biosensors.

**Figure 1:**
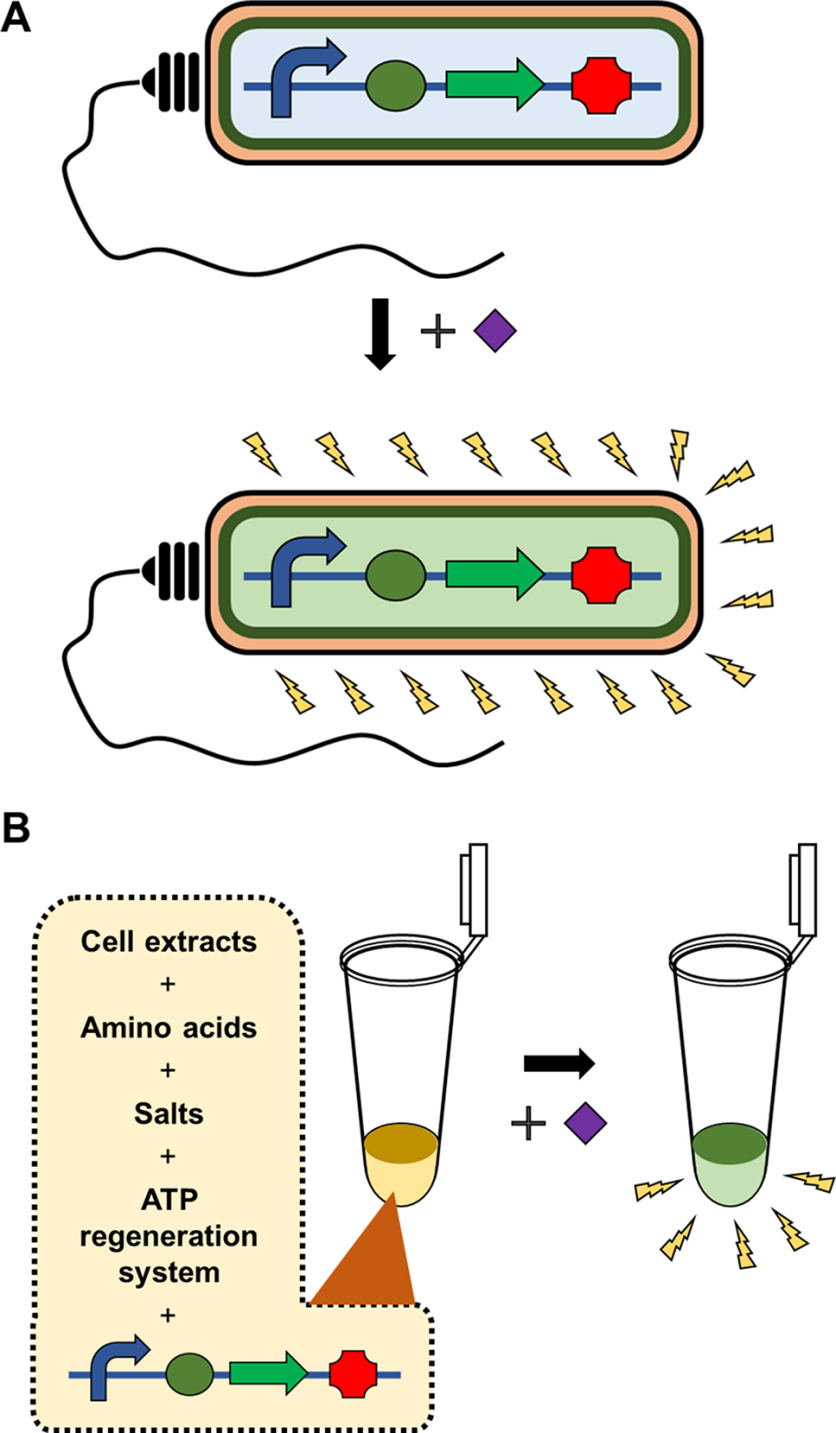
Whole cell-based microbial sensors vs cell-free biosensors. Microbial biosensors require viability and integrity of the cells harboring the gene constructs to detect and report the presence of target analytes, which substantially limits the variety, detection level, and detection range of target analytes **(A)**. In contrast, cell-free biosensors harness the cellular synthetic machinery after extracting it from the cells. This *in vitro* nature of cell-free biosensors provides far greater flexibility, stability and operational convenience of biosensors **(B)**.

Recently, an increasing number of studies have employed cell-free protein synthesis (CFPS) as an alternative tool for producing recombinant proteins (Catherine et al., 2013; Zemella et al., 2015; Schinn et al., 2016; Dopp et al., 2019). Since Nirenberg and Matthaei (1961) first demonstrated that *Escherichia coli* (*E. coli*) extract can be programmed with nucleic acids to produce encoded proteins, CFPS systems have continuously evolved to improve productivity and operational convenience (Spirin, 2004; Kim et al., 2007; Jewett et al., 2008; Kim et al., 2008; Cai et al., 2015; Rues et al., 2017; Jaroentomeechai et al., 2018). As a result of those efforts, the present CFPS systems can now readily produce mg/ml of recombinant proteins (Kim et al., 2007; Jewett et al., 2008). By using cellular synthetic machinery extracted from viable cells, a CFPS system provides a complete set of the machinery required for protein expression without requiring intact cellular structure and viability. This, in turn, allows instant biosynthesis of proteins upon addition of template DNA to a homogeneous reaction mixture. More importantly, the open nature of CFPS allows direct access to the individual steps of protein synthesis, for example, transcription of template DNA, aminoacylation of tRNA, and translation of mRNA. Each of these modularized steps can be the target for engineering to make the process of protein synthesis dependent upon the presence of specific compounds. Due to these unique features, CFPS can be successfully implemented as a biosensor, avoiding many of the problems associated with whole cell-based microbial biosensors (**Figure 1**).

Here, we review recent advances in the development of biosensors that harness the amplifying nature of cellular synthesis, including the processes of amino acylation, transcription, and translation. In a recent review, Soltani et al. (2018) surveyed the studies to develop cell-free biosensors, categorized according to the targeted stage of protein synthesis. While the previous review focused on the mechanisms for production of signal-generating proteins during cell-free biosensing, here, we focused on mechanisms to trigger cell-free synthesis of signal-generating indicators using diverse molecules as recognition elements, including transcription factors, aminoacyl tRNA synthetases, toehold switches, and aptamers.

## Target Recognition Based on Nucleic Acid Hybridization

Detection of specific nucleic acids (DNA or RNA) comprises the basis of molecular diagnostics. In the case of infectious diseases, nucleic acid-based diagnostics detect DNA or RNA from the infecting organism. For non-infectious diseases, nucleic acid-based diagnostics may be used to detect a specific gene or mutation, or dysregulated expression of a gene associated with disease. Among currently available assay methods, PCR has been established as the gold standard platform for detection of nucleic acids. A major advantage of PCR-based methods is the ability to exponentially amplify the target sequences, enabling rapid detection of targets present in very low concentrations. However, PCR-based detection methods require sophisticated laboratory facilities, expensive equipment, and well-trained operators. This substantially limits the use of PCR at the point of care for routine monitoring of patients. The availability of readily accessible and portable diagnostic methods is especially in demand for disease control in resource-poor regions. Biosensors that can easily detect target nucleic acids at an affordable cost will contribute to the management of public health and environmental monitoring. Because the specificity of nucleic acid detection relies on base pairing between two complementary strands, the recognition element of a non-PCR biosensor should also be based on the hybridization of sensing nucleic acids with target nucleic acids.

**Figure 2:**
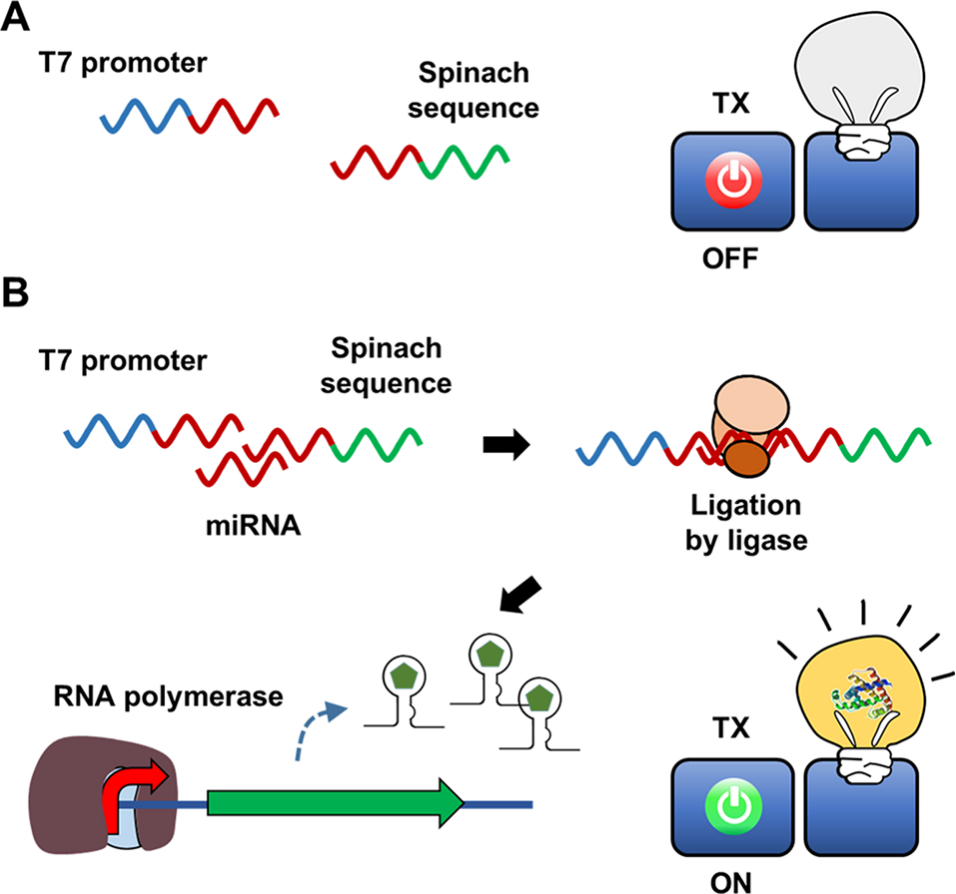
Hybridization-dependent formation of signal DNA. In the scheme developed by Ying et al., sequences of the T7 promoter and the Spinach gene are added in separate DNA strands, and thus the RNA polymerase cannot transcribe the Spinach aptamer **(A)**. Target nucleic acid sequence in the assay sample, however, serves as a structural support to enable ligation of the two DNA strands **(B)**. Therefore, the Spinach aptamer is produced to give fluorescence signal only in the presence of the target nucleic acid.

In addition to acting as a template for protein synthesis (mRNA), certain species of RNA, such as tRNA and miRNA, carry out important biological functions in the absence of translation due to the ability of RNA to form secondary structures through intramolecular base pairing. Furthermore, the advent of systematic evolution of ligands by exponential enrichment (SELEX) technology has enabled screening of synthetic RNAs from a combinatorial library for the desired functions (Ellington and Szostak, 1990; Tuerk and Gold, 1990). Spinach is a synthetic RNA molecule derived by SELEX technology. Spinach is an RNA aptamer that binds 4-hydroxybenzlidene imidazolinone (HBI), a compound that mimics the spontaneously modified form of the tripeptide Ser65-Tyr66-Gly67 from GFP (Paige et al., 2011). Upon binding with Spinach, HBI emits strong fluorescence and thus can visualize the entrapped RNA. Ying et al. (2018) developed a Spinach-based cell-free sensor in which *in vitro* transcription of the Spinach gene was triggered by specific miRNAs in assay samples (**Figure 2**). To detect the presence of miRNA and trigger Spinach synthesis, Ying et al. used two separate single strands of DNA containing the T7 promoter and Spinach sequences. Because these DNA strands were designed to be partially complementary to the target miRNA, the miRNA in the assay sample serves as a structural support to enable ligation of the two DNA strands. The resulting single-stranded DNA allows T7 RNA polymerase to transcribe the Spinach gene downstream of the T7 promoter in an *in vitro* transcription reaction. The detection of limit of the Spinach-based system was 3 pM for miRNA. In a subsequent study, Tang et al. (2018) further advanced this system by using a tandem Spinach sequence to enhance miRNA detection sensitivity.

In addition to Spinach, RNA aptamers with different fluorescence properties have been developed (Dolgosheina et al., 2014; Filonov et al., 2014), which can be combined with the same sensing mechanism to provide more flexibility and multiplexity in RNA-based biosensing. This approach of using transcripts as a signal-generating molecule allows for simple and convenient preparation of biosensors. However, this method only partially harnesses the cascading amplification power of gene expression (DNA to RNA to protein), and it may be more advantageous to combine target-triggered transcription with a subsequent translation reaction to obtain stronger signal readouts.

**Figure 3:**
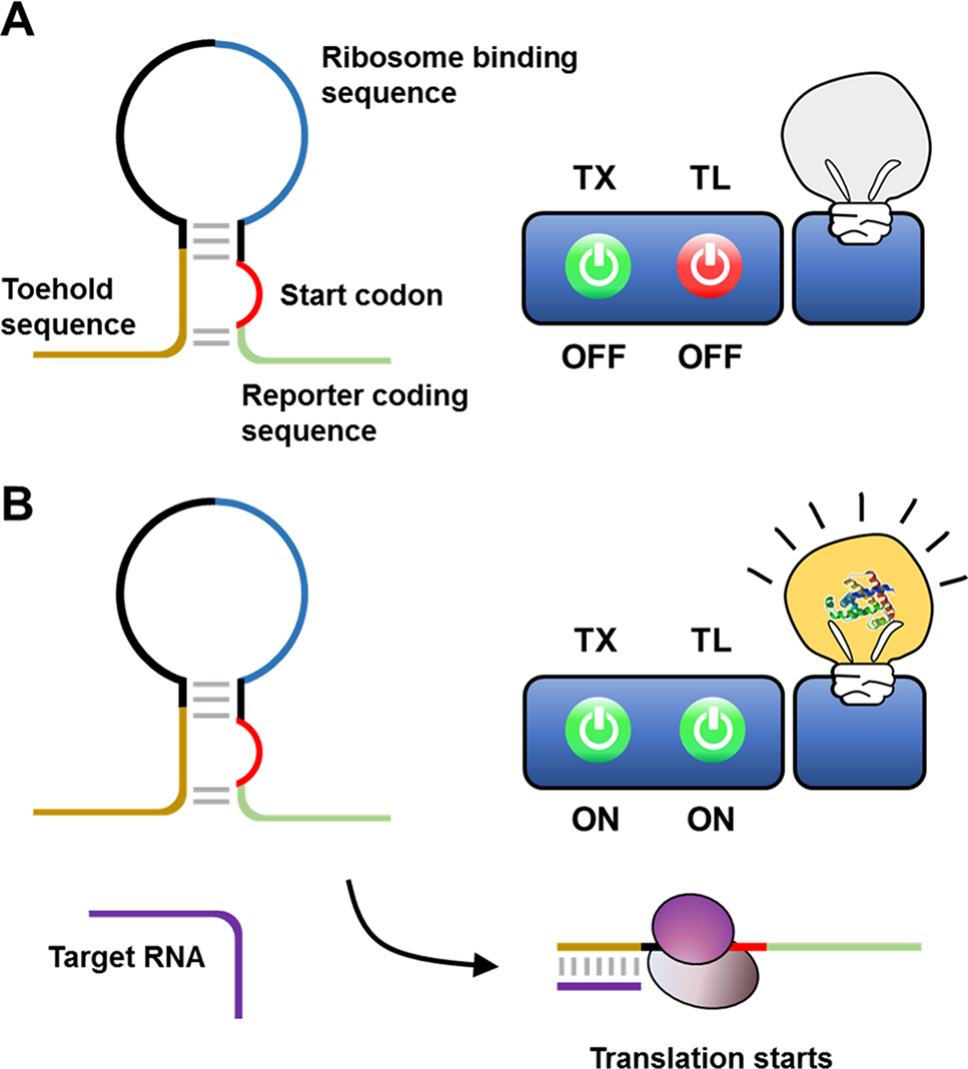
Cell-free biosensor employing a toehold switch as a recognition element. Ribosomes cannot translate a reporter protein due to the limited accessibility to ribosome binding sequence and the start codon **(A)**. Hybridization of the target nucleic acids with the toehold switches, however, expose the ribosome binding sequence and the start codon for ribosome to initiate the translation **(B)**.

Indeed, Pardee et al. (2016) proposed a streamlined cell-free expression biosensor for sensitive detection of Zika virus RNA (**Figure 3**). After amplifying a pre-defined region of Zika RNA through an isothermal nucleic acid sequence-based amplification (NASBA) reaction, amplified RNA segments were applied to the reaction mixture, causing cell-free translation to trigger synthesis of a reporter protein by turning on a toehold switch. The toehold switch was designed to sequester the ribosome-binding sequence and start codon of the reporter mRNA in a hairpin loop. Translation of reporter mRNA is triggered by hybridization of the toehold switch with Zika RNA, which exposes the ribosome-binding sequence such that it becomes accessible to ribosomes. Zika RNA was successfully detected at a threshold of 2.8 fM using this cell-free biosensor. When equipped with a novel CRISPR/Cas9-based module, this system can discriminate between viral strains with single-base resolution. They demonstrated the rapid development of a diagnostic workflow for sequence-specific detection of pathogen nucleic acids, addressing current limitations in the practical application of molecular diagnostics by combining isothermal RNA amplification with a toehold switch cell-free biosensor.

## Transcription Factor-Based Recognition Elements

**Figure 4:**
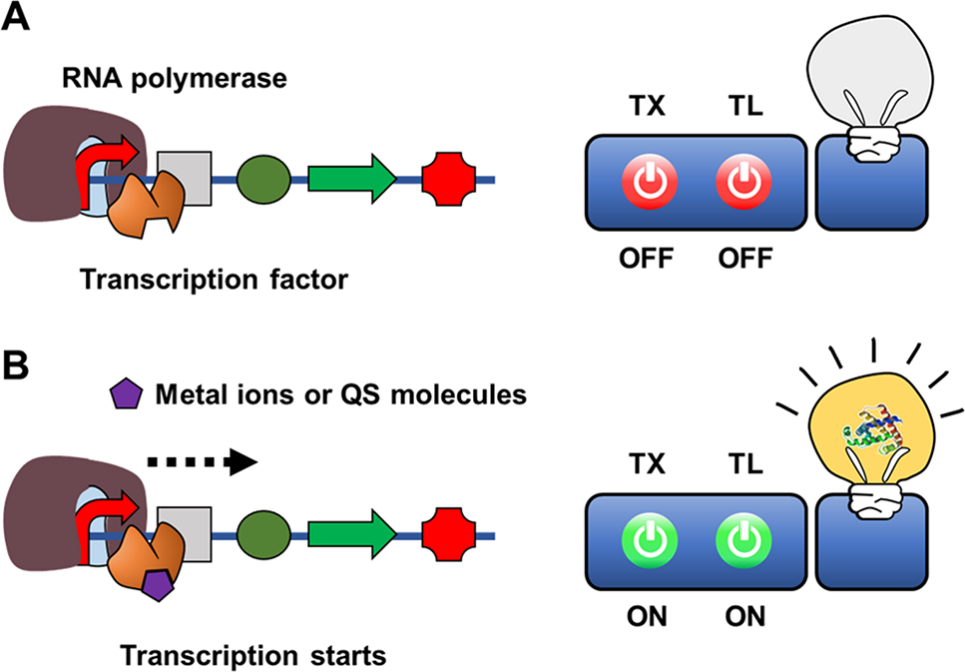
Cell-free expression biosensor employing a transcription sensor as a recognition element in the absence **(A)** or presence of **(B)** target molecules.

To physiologically adapt to changing environment and growth conditions, living organisms have evolved sensing systems to detect diverse chemical compounds. Transcription factors play an important role in controlling microbial physiology at the transcriptional level, regulating the expression of cellular proteins in response to specific chemical compounds (Daunert et al., 2000). Effector-triggered transcription factor regulation of gene transcription can be utilized *in vitro* to develop a sensing mechanism for cell-free biosensors to detect chemical molecules (**Figure 4**).

While some metal ions serve as essential cofactors for cellular enzymes, many other metal ions are highly toxic to viable cells. To maintain viability, microorganisms have highly sensitive discriminatory mechanisms to remove or neutralize toxic metal ions such as mercury, lead, and cadmium. For example, MerR family transcriptional activators function as regulators of Gram-negative mercury resistance (mer) operons that encode a mercury transporter protein and mercury reductase (Brown et al., 2003). Upon binding with a mercury ion, MerR activates operon transcription. Pellinen et al. (2004) developed a cell-free biosensor for detection of mercury ions using MerR as a recognition element. They constructed a sensor plasmid that encodes firefly luciferase under control of the mer operator-promoter, such that MerR-mediated transcriptional activation regulates synthesis of firefly luciferase. When the CFPS reaction mixture containing sensor plasmids and purified MerR was spiked with an HgCl_2_-containing assay sample, HgCl_2_ was successfully detected, with a detection range of 1–10,000 nM. When compared with a whole-cell biosensor using the same DNA construct, the cell-free biosensor provided a much wider detection range (1 to 10,000 vs 2 to 200 nM). A similar approach was adopted for detection of quorum-sensing (QS) molecules from pathogenic microorganisms. Kawaguchi et al. (2008) developed a cell-free expression biosensor capable of detecting the N-acyl homoserine lactone (AHL) class of QS molecules. To build a cell-free biosensor for AHL, they first prepared cell-free extracts from *Agrobacterium tumefaciens*, which produces TraR, an AHL-dependent transcriptional activator. The sensor plasmid in the reaction mixture consists of a lacZ coding sequence downstream of the TraR-binding sequence (traI), such that addition of AHL produces β-galactosidase to generate a chromogenic signal. The detection limit of AHL by this cell-free biosensor was 100–300 nM.

Similarly, to detect the γ-butyrolactone class of QS molecules, Yang et al. (2009) developed a cell-free expression biosensor employing the heterologous transcription system of *Streptomyces coelicolor*. They prepared cell-free extract from *E. coli* BL21(DE3) supplemented with the γ-butyrolactone receptor ScrbR, which was derived from *Streptomyces coelicolor* and served as a recognition element. The sensor plasmid encodes a scbR/A-binding site between the T7 promoter and eGFP. Using this cell-free biosensor, they examined various QS molecules of other *Streptomyces* species and Gram-negative bacteria at concentrations ranging from approximately 4 to 80 μM. Collectively, these studies demonstrated that QS molecules such as N-hexanoyl-DL-homoserine lactone can initiate crosstalk between Streptomyces and Gram-negative bacteria.

Chappell et al. (2013) developed a cell-free expression biosensor to detect 3O-C12-HSL, which is a QS molecule used by *Pseudomonas aeruginosa*, a bacterial species often contracted by patients with cystic fibrosis. This system utilized GFP as the reporter protein, and LasR as the recognition element. Wen et al. (2017) demonstrated that the CFPS biosensor developed by Chappell et al. (2013) was more effective than the corresponding cell-based biosensor in clinical samples, demonstrating greater specificity for the QS molecule, less cross-activation by non-target QS molecules, and a lower detection limit (5 vs 22 nM 3O-C12-HSL).

## Aptamer-Based Target Recognition

Although transcription factor regulation of transcription serves as a useful mechanism for target recognition in a number of cell-free biosensors, many desired analytes cannot be detected by any known transcription factors, limiting the scope of this approach. Synthetic nucleic acid aptamers are an alternative option to design a recognition element for cell-free biosensors. Because aptamers can potentially be selected against any ligand of interest from a combinatorial library by SELEX, if the event of aptamer-ligand binding is linked with regulation of gene expression, this approach will provide more flexibility in the design of cell-free biosensors.

**Figure 5:**
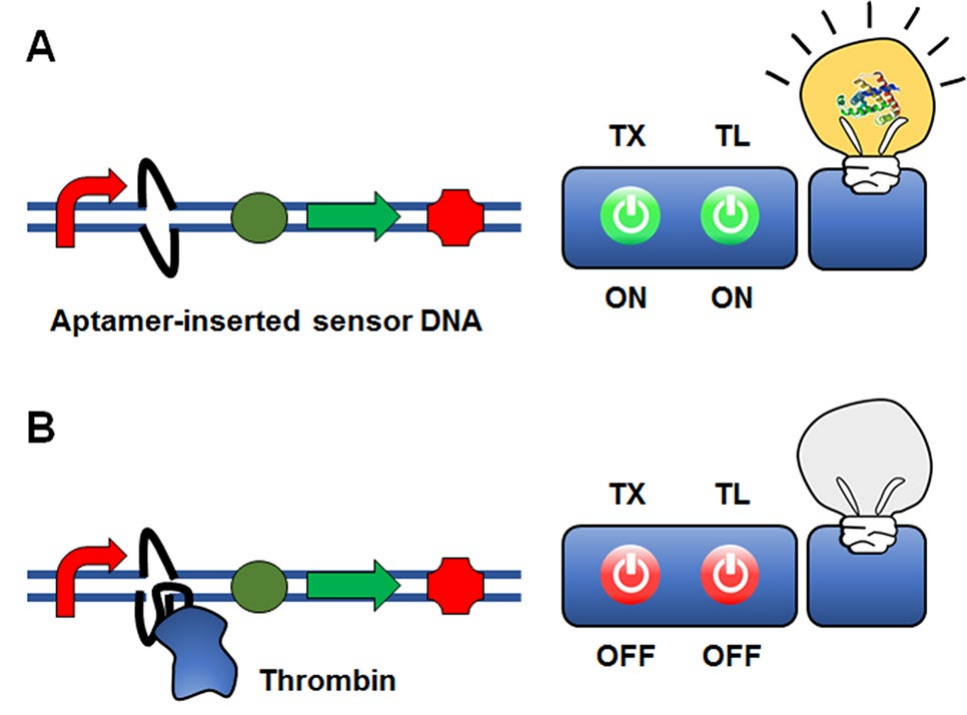
Cell-free expression biosensor employing a single-strand aptamer as a recognition element. An aptamer sequence that binds the target molecule is embedded in the reporter gene construct. Although the presence of the aptamer sequence is permissible for transcription **(A)**, the aptamer-target binding causes the formation of a ‘bubble’ DNA region. This restricts the binding of the RNA polymerase to the promoter, and thus switches off transcription **(B)**.

Iyer and Doktycz (2014) suggested an interesting approach of using an aptamer sequence to control gene expression at the transcriptional level (**Figure 5**). In this approach, an aptamer sequence that binds the target molecule is embedded in the reporter gene construct. As a proof-of-concept model, they inserted a thrombin-binding ssDNA aptamer sequence downstream of the T7 promoter. Upon thrombin binding, a ‘bubble’ DNA region is formed, such that thrombin binding to the aptamer sequence inhibits transcription of T7 RNA polymerase through steric hindrance. The insertion of the aptamer sequence proximal to the T7 promoter sequence generated a 6-fold change in GFP reporter gene expression when thrombin was present at a relatively high concentration compared with conventional thrombin detection methods. Although the sensitivity for thrombin detection needs to be improved, this proof-of-concept study demonstrated that protein molecules can be detected by an aptamer-based ligand-binding system rather than a conventional operator-transcription factor regulatory system.

Aptamer-mediated transcriptional control was further improved by introducing a different means of transcriptional regulation. Wang et al. (2017) developed a method that employs duplex aptamers upstream of the T7 promoter in both the sense and antisense strands of a double-stranded plasmid. In this plasmid construct, the non-complementarity between the DNA aptamers causes formation of a bubble structure. The DNA bubble is further enlarged when the ligand binds aptamers on both sides of the bubble structure, increasing RNA polymerase access to the downstream promoter region. Therefore, the presence and amount of thrombin is reflected by enhanced production of reporter protein. Although the sensitivity of this approach needs to be further improved, these results clearly demonstrate the potential of using aptamers as recognition elements in cell-free biosensors.

## Use of Translational Machinery as a Recognition Element

**Figure 6:**
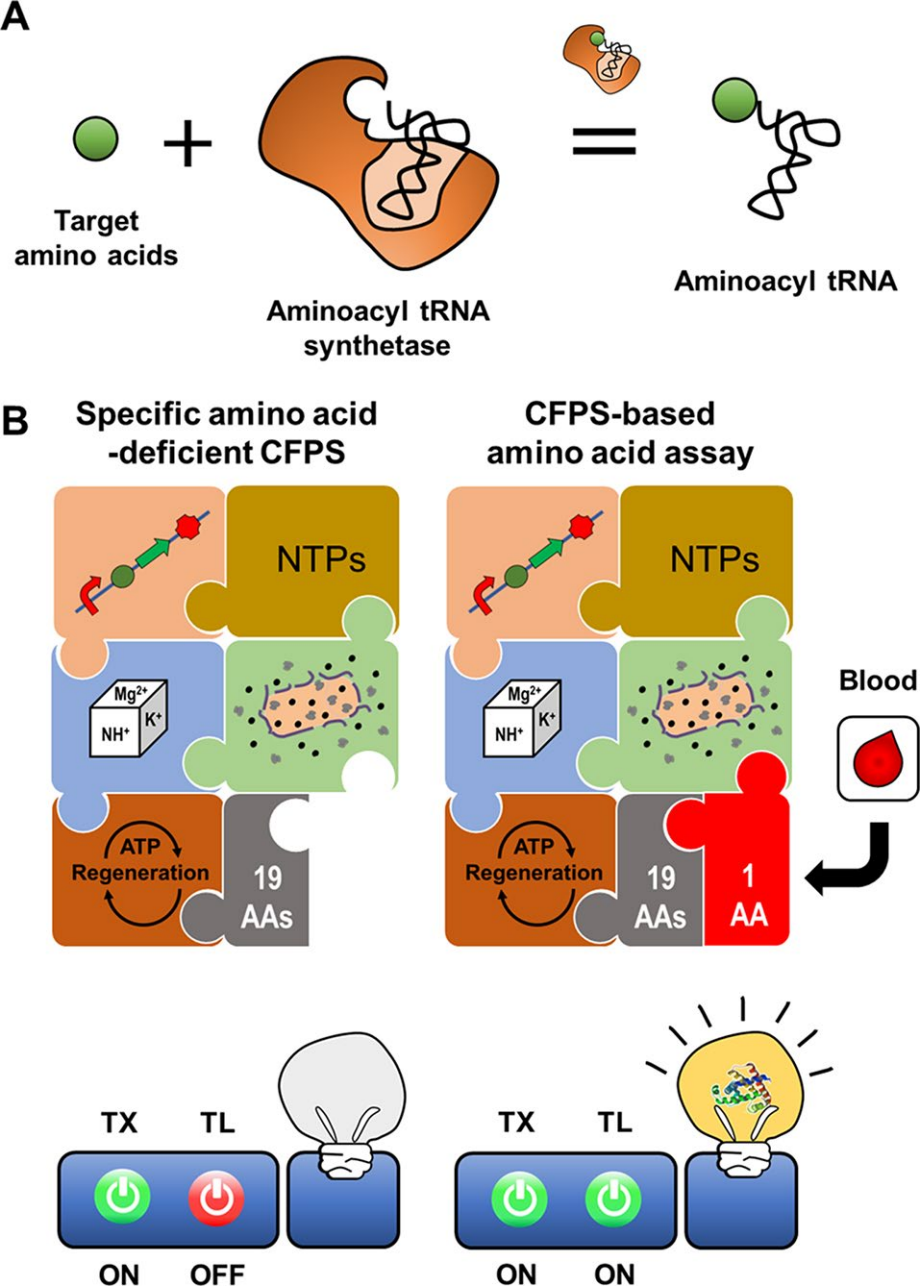
Cell-free expression biosensor employing an aminoacyl tRNA synthetase as a recognition element. Aminoacyl tRNA synthetase provides ribosome with charged tRNA **(A)**. The reaction mixture for CFPS missing an amino acid cannot produce the reporter protein due to the absence of the charged tRNA. The incomplete reaction mixture resumes the synthesis of a reporter protein when the reaction mixture is supplemented with an assay sample containing the missing amino acid **(B)**.

Jang et al. (2017) demonstrated that aminoacyl tRNA synthetase (AARS) can be used as the recognition element for cell-free biosensors (**Figure 6**). Amino acid analysis is important for a diverse range of fields including food science, environment science, pharmaceuticals, and diagnostics (Leuchtenberger et al., 2005; Kimura et al., 2009; Otter, 2012; Yatabe et al., 2013). For example, elevation of certain plasma amino acids in humans may indicate metabolic disease. Recent reports have identified that the plasma amino acid profile may be closely related to specific cancers and diabetes (Kimura et al., 2009). Presently available methods for amino acid analysis require complex laboratory setups and expensive equipment (Zytkovicz et al., 2001; Kaspar et al., 2009; Le et al., 2014). Development of simple and easy analytical methods for point of care diagnostics with conventional approaches such as liquid or gas chromatography is therefore prohibitive. Jang et al. (2017) developed a simple and economical cell-free expression biosensor for amino acid quantification that exploits translational machinery, and is based on the principle that amino acids are the essential building blocks for protein.

A CFPS reaction mixture devoid of specific components, for example, certain amino acids, can be activated to produce protein outputs upon exogenous supplementation of the missing components. The components of CFPS can be selectively removed from the reaction mixture to make protein synthesis dependent on exogenous addition of these components, for example, amino acids in a patient plasma sample (Catherine et al., 2015). The cell-free expression biosensor for amino acid detection harnesses an amino acid-deficient CFPS system as a conditional signal-generating module, in which reporter proteins are generated when the reaction mixture is supplied with specific amino acids (Jang et al., 2017). When mixed with assay samples containing the deficient amino acids, incubation of the cell-free synthesis reaction mixture rapidly produces the reporter protein, superfolder GFP (sfGFP). The sfGFP fluorescence intensity is representative of the actual amino acid concentrations in the assay samples. A crude extract-based cell-free expression biosensor effectively detected 16 amino acids such that concentration of sfGFP production corresponded linearly to the amino acid concentration from 0.1 to 100 μM. However, the system was unable to detect Asp, Asn, Glu, and Gln, which generate background signal due to reversible conversion by metabolic enzymes in crude extracts. For these exceptional amino acids, the PURE CFPS system, which includes a minimal set of individually purified proteins and cofactors needed for transcription and translation, was effective, with a detection threshold of 20 nM. This cell-free expression biosensor accurately detected fetal bovine serum amino acid content, yielding results almost identical to those of the traditional high-performance liquid chromatography assay. Although the PURE system is costly to produce, it is suitable for amino acid detection without background signals or interference derived from undesirable reactions. Nevertheless, the crude extract-based CFPS system has the potential to detect amino acid metabolites derived from amino acid metabolism in cell extracts.

## Interfacing Cell-Free Synthesis With Existing Methods for Target Recognition

**Figure 7:**
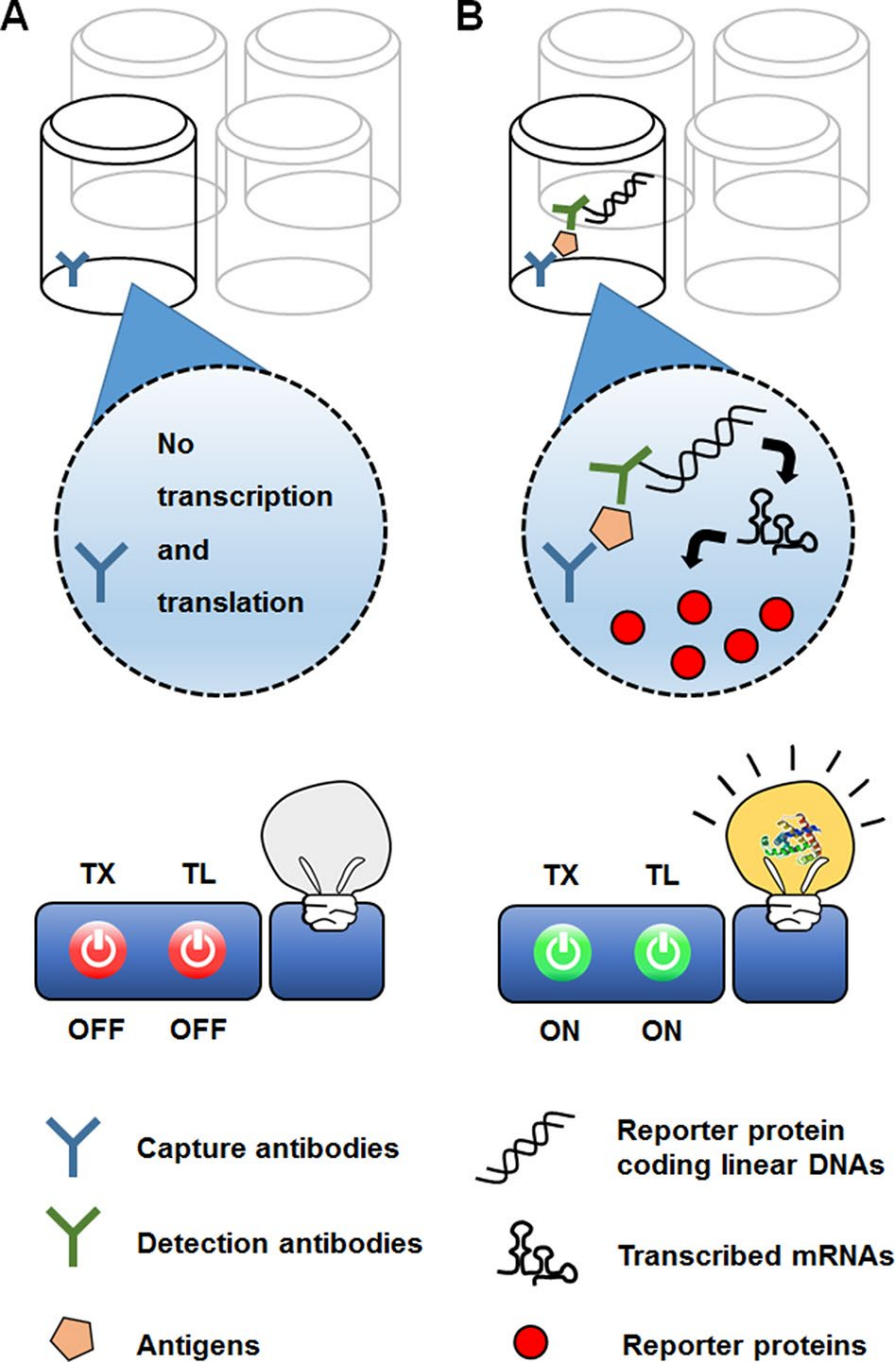
Use of CFPS as a signal amplifier in an immunoassay. In the expression immunoassay, instead of a signal-generating enzyme, DNA sequence encoding the enzyme conjugated to a detection antibody. While the reporter-encoding DNA is washed off the absence of target analyte **(A)**, addition of the anatlye allows the DNA to be anchored on the surface of microtiter well **(B)**. The DNA on the target-bound detection antibody is then translated into multiple molecules of the signal-generating enzyme, thereby increasing the sensitivity of detection.

In a different approach to *in vitro* use of the translational machinery for analytical purposes, CFPS can be combined with previously established biosensing platforms. For example, CFPS can be interfaced with the enzyme-linked immunosorbent assay (ELISA) technique (**Figure 7**). In the conventional sandwich configuration, an ELISA uses a detection antibody to recognize target molecules immobilized by a capture antibody. The detection antibody is conjugated with an enzyme that generates chromogenic, luminescent, or fluorescent signals. The ELISA sensitivity is thus determined by the catalytic efficiency of the enzymes conjugated to the detection antibody (Schuurs and van Weemen, 1980). In an alternative approach originally developed by Christopoulos et al. (1995), the signal-generating enzyme on the detection antibody was replaced with DNA encoding the enzyme. The DNA on the target-bound detection antibody was then transcribed and translated by adding the reaction mixture for CFPS. Introduction of the additional step in this new method, known as an expression immunoassay, substantially improved the sensitivity of detection by combining the high specificity of the ELISA with the protein-amplifying power of cell-free synthesis. However, this method has not been widely accepted, mainly due to the poor reproducibility and low translational efficiency of the rabbit reticulocyte lysate used to generate the CFPS reaction mixture.

Byun et al. (2019) improved the reproducibility and sensitivity of the expression immunoassay by introducing *E. coli* extract as an efficient translation module to convert the antibody-conjugated DNA into reporter enzymes. By optimizing reaction conditions for cell-free synthesis of antibody-conjugated DNA, they markedly improved the yield of enzyme synthesis in an expression assay for alpha-fetoprotein, yielding a detection threshold as low as 7 fM. This is approximately a 100-fold lower limit of detection than that of the original expression immunoassay employing the rabbit reticulocyte lysate-based CFPS. However, implementing an amplification step by a CFPS also adds extra processing time to the already time-consuming ELISA. For the expression immunoassay to become a more viable ultrasensitive immunoassay option, the number of steps should be minimized, and the time for cell-free expression of antibody-conjugated DNA should be shortened.

**Figure 8:**
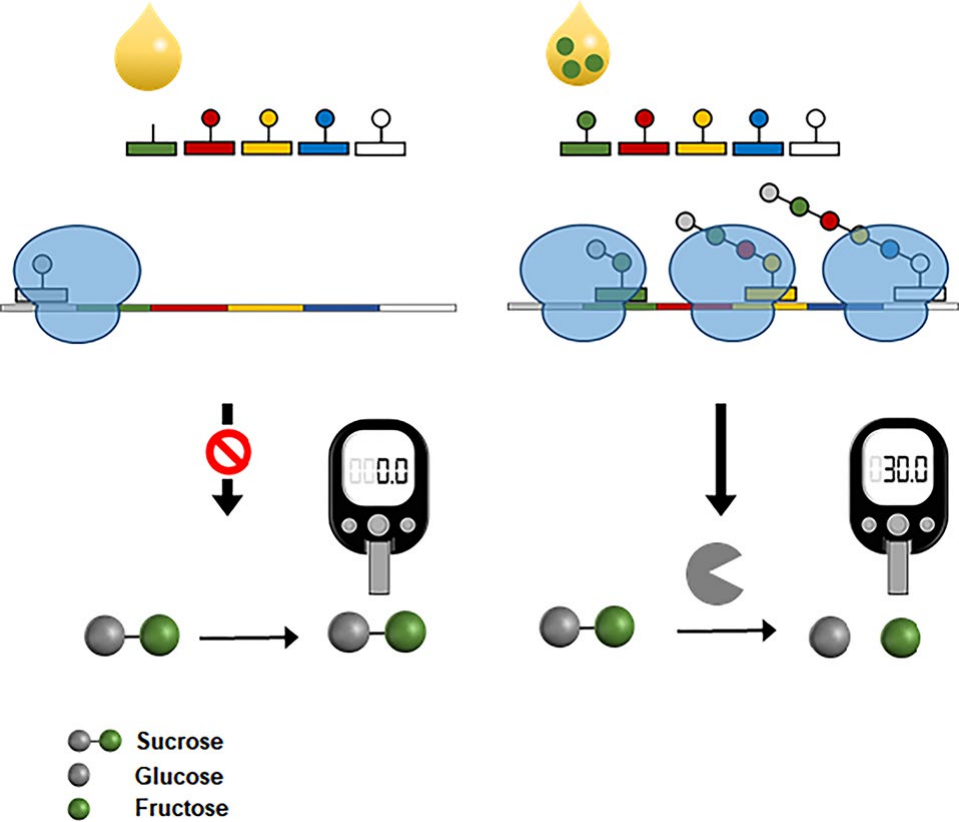
Interfacing CFPS with a personal glucose meter for amino acid measurement. The reaction mixture for CFPS is programmed to produce a bacterial invertase when complemented with the target amino acids from samples. Expression level of invertase is proportional to the concentrations of amino acids in samples. Cell-free synthesized invertase generates glucose molecules from sucrose, which can be measured by a personal glucose meter.

More recently, CFPS was also successfully interfaced with the widespread personal glucose meter to measure amino acids (Jang et al., 2019). In the same principle used for the fluorescent detection of amino acids (Jang et al., 2017), the reaction mixture for CFPS was prepared devoid of specific amino acids, which were the target analytes. The reaction mixture was programmed to produce bacterial invertase when complemented with the missing amino acids. Upon the addition of assay samples containing the target amino acids, functional invertase was produced in proportion to the amino acid concentration. Because the expression level of invertase could be readily determined by measuring the glucose it generated from sucrose, this system enabled measurement of amino acid concentration with a personal glucose meter (**Figure 8**). This result demonstrates the possibility of harnessing CFPS as a converter to measure diverse analytes using the readily accessible personal glucose meter.

## Conclusions

Maintenance of extremely complex biological systems relies on the specific interactions of biomolecules. The high specificity of biomolecules has therefore been harnessed by numerous biosensors to probe target analytes. However, accurate target recognition is necessary but not sufficient for successful biosensing, as the binding event between the recognition element and target analytes must be converted into a measurable signal by a transducer. The amplifying nature of the gene expression process can be harnessed to construct a genetically encoded transducer that converts binding of target analytes into measurable signals.

As summarized in **Table 1**, the results surveyed in this review demonstrate that cell-free protein synthesis can be usefully implemented to generate signals in response to various analytes. However, a number of issues need to be addressed for widespread use of cell-free biosensors. First, diverse recognition elements need to be developed for cell-free generation of signal molecules in response to various types of analytes. Second, it might be required to engineer the recognition elements to improve the specificity and sensitivity of cell-free biosensors. Third, it is highly desirable to develop cell-free synthesis systems that can produce signal molecules that are commonly used in generic bioassay methods. For example, a cell-free synthesis system that can produce functional horse radish peroxidase will be readily implemented into the existing assay platforms to enhance the yield of signal amplification.

**Table 1:** Comparison of different biosensing methods based on a cell-free protein synthesis system.

Recognition elements	Target analytes	Signal generating elements	Detection range	References
mRNA (Toehold switch)	Zika virus RNA	Beta-galactosidase	3 fM-30 pM	Pardee et al. (2016)
Transcriptional regulator (MerR)	HgCl_2_	Firefly luciferase	1-10000 nM	Pellinen et al. (2004)
Transcriptional regulator (TraR)	N-acyl homoserine	Beta-galactosidase	100-300 nM	Kawaguchi et al. (2008)
Transcriptional regulator (ScbR)	Gamma-butyrolactone	Enhanced GFP	4-80 uM	Yang et al. (2009)
Transcriptional regulator (LasR)	N-acyl homoserine	GFP	1-100 nM	Chappell et al. (2013)
DNA aptamer	Thrombin	GFP	>1 μM	Iyer and Doktycz (2014)
DNA aptamer	Thrombin	GFP	0.2-1.2 μM	Wang et al. (2017)
Aminoacyl tRNA synthetase	Amino acids	Superfolder GFP	0.1-100 μM	Jang et al. (2017)
Aminoacyl tRNA synthetase	Amino acids	Invertase	0.1-20 μM	Jang et al. (2019)
Antibody conjugated with DNA	alpha-fetoprotein	Firefly luciferase	7 fM-36.2 pM	Byun et al. (2019)

As progress in synthetic biology provides a rapidly increasing number of novel and efficient components that can be readily implemented for target recognition and signal generation, the application of cell-free biosensors will continue to grow in diverse fields, including pharmacological diagnostics and biotechnology.

## Author Contributions

In collaboration, K-HL and D-MK wrote all sections of this manuscript. D-MK perceived the significance of the topic and drafted the manuscript. K-HL drafted the manuscript, made substantial contributions to the acquisition, analysis, and interpretation of data, and illustrated the figures.

## Funding

This work was supported by the National Research Foundation of Korea (grant numbers 2014M3C1A3051473 and 2016M1A5A1027465).

## Conflict of Interest

The authors declare that the research was conducted in the absence of any commercial or financial relationships that could be construed as a potential conflict of interest.
